# The Institutional Library

**Published:** 1918-08-24

**Authors:** 


### THE INSTITUTIONAL LIBRARY.

### MOTHER AND CHILD.

**The Mother and the Child.** By William S. Sadler, M.D., and Lena K. Sadler, M.D. (London: George G. Harrap and Co. 1918. Pp. 456 + x. Price 6s. net.)

The inspiration of this book, it appears, is in part professional and in part parental. The cry for it has come, we are told, alike from patients, public, and publishers, and the authors being, as we learn from the announcement, "parents as well as skilled physicians," have answered to the "insistent call." Certainly there is no stint in the information supplied, but whether it is necessary or even advisable that "everyone who has to do with the care of the child" should acquire all this information may be doubted. An instance of the elaborate and detailed fashion in which the various subjects are treated may be found in the section dealing with anæsthesia in childbirth. Here we have the claims of "twilight sleep" set out in no less than thirteen numbered paragraphs; next come the "dangers" of the method in eleven paragraphs; and finally the "conclusions" of the authors in eighteen paragraphs. Yet after having attempted to digest this agitating debate, the expectant mother will find that there is a still better method of avoiding the pains of childbirth—namely, the inhalation of nitrous oxide gas, poetically named for this particular purpose "sunrise slumber," and the virtues of which are exhausted in twelve paragraphs. The child is not less liberally treated than the mother, as is seen in the fact that each of the twelve fashions of crying included among its capacities receives the recognition of a diagnostic description. For our own part we think all this argument and debate ill-suited for lay minds, and while we recognise its thoroughness we cannot accept Dr. and Mrs. Sadler's volume as well suited to the audience it proposes to address.

**Painless Childbirth in Twilight Sleep in the East.** By Cecil Webb-Johnson, M.B., R.A.M.C. (London and Calcutta: Butterworth and Co. 1918. Pp. 123+vi. Price 4 rupees net.)

"Twilight sleep" has become the battleground of parties and the signal of an acute controversy. Denounced by an eminent London obstetrician as a "fraud," it has been highly commended by a not less distinguished Edinburgh teacher; whilst discussions on the subject at various societies have betrayed much animation. The phrase and the practice have not been altogether happy in their friends, for articles in Sunday papers and photographs of mothers and babies are not the most likely means to secure a calm professional judgment. In these circumstances the recorded personal experiences of a careful observer are valuable contributions, and it is in this classification that Captain Webb-Johnson's book must be placed. He modestly suggests that in America and England the cause has advocates whom he cannot expect to rival, but he trusts to be effective in India and the East. Personally, we have never found the argument more successfully presented than in his book, and we will venture to offer him a compliment on the literary quality of his pages. He tells us what he himself has seen and done, and he does this in a most engaging fashion. The book has a brief "foreword" by Captain V. B. Green-Armytage, M.D.

**How to Enlighten our Children.** A Book for Parents. By Mary Scharlieb, M.D., M.S. (London: Williams and Norgate. 3s. 6d.)

The question of sex instruction has lately come into increased prominence among educationists and practical teachers and the more thoughtful among parents. The advisability of definite teaching at school or at home has been earnestly debated at conferences. At the University Extension meeting at Oxford last August Dr. Stephen Paget gave to an audience of both sexes and all ages the address which he has since published advising an organised family ceremony of initiation for each child on reaching a suitable age. The sympathy of the audience was distinctly with Dr. Paget on this occasion. Undoubtedly many parents will have to overcome a sense of reluctance to treat frankly of knowledge which they fear may brush the bloom from young souls, even if they realise that much of the physical and spiritual evil and suffering of our civilisation are due to an ignorance that is not necessarily innocence. If this fence is crossed they are confronted by another: the difficulty of knowing what to say to girl or boy, and how to say it. Indeed, many mothers have to remember that a not inconsiderable amount of preventible distress in their own lives was due to ignorance of physiology. On all grounds this excellent manual should be most welcome to all to whom the subject appeals. The biology and physiology involved in the transmission of life are simply and clearly stated, not without consideration of the psychology of the adolescent, and the wisest possible counsels given for the initiation of girl and boy into the mysteries of their own being. The wise care of health without undue fuss is encouragingly expounded, and chapters on the social evil and an unexaggerated consideration of eugenics complete a most valuable book.

### THE BABIES' GOSPEL.

**Natural Feeding of Infants.** By F. Truby King, C.M.G., M.B. Introduction by J. S. Fairbairn, M.D. (Whitcomb and Tombs. 1s. net.)

Dr. Truby King's propaganda of the Babies' Gospel of Natural Feeding has now been crystallised into a small but none the less important brochure. Some of his statements, the result of twelve years' practical experience at his Mother and Child Hospital, Dunedin, N.Z., will still be new and startling to many persons in this country. Thus the theory that breast-feeding can be restored after a week, a fortnight, or even a longer time of weaning, and the supply made more than adequate, has been abundantly proved, and the nursing maintained after the return to home conditions. In case of infectious disease it has been shown that the milk can be drawn off and nursing satisfactorily resumed when infection is past. Even maternal syphilis becomes only another reason for not denying the baby its just right, and if or while the supply is inadequate the breast milk should be given at each feed, and cow's milk as a second course to make the

**The Institutional Library**—*(continued)*.

full amount. Dr. King has a gospel for mother as well as for babe. Congestion and falling of the organs he considers frequently to be directly due to weaning, the natural blood-supply being diverted from its intended purpose, whereas nursing produces a natural shrinkage. But the strain upon the mother caused by two or two-and-a-half hour feeds as well as one during the night is needless, and babies are often made to suffer by this over-feeding. Three to four hour feeds were recommended by Dr. Thomas Bull in the middle of the last century, and a properly nourished child should if it wakes in the night be given only a little water. This, indeed, it may sometimes need during the day, especially in hot weather. An interesting illustration of the advantage in all development—for instance, of hair, teeth, etc.—of natural feeding is found in the description of "Paris calf" boots made from the skin of young animals reared by their mothers. The leather is astonishingly superior to that of the "bucket-fed."

**The Baby.** By E. ARTHUR SAUNDERS, M.A., M.B., F.R.C.P. (London: Methuen. 1s. 3d. net.)

DURING the past three and a half years the lethargy which for too long had characterised our attitude towards the problems of child-life has been gradually broken up, and to-day thought, both medical and lay, is being directed to the subject with an ever-increasing earnestness and intensity. And the problem is not one that can be settled by Select Committees and Commissions. It is essentially one that needs intelligent co-operation between the scientist and the ordinary mothers of our people. For this reason we welcome the book before us. It is not a scientific argument, but a practical exposition based upon scientific knowledge, written by one who has made a special study of children and who gives in clear and simple language invaluable guidance to both mothers and nurses. We cannot summarise the book better than by saying it summarises the baby and his care from birth till he is old enough to burn and choke himself. The first fatal year is fully dealt with, and no mother can submit herself to Dr. Saunders' guidance without her child deriving inestimable benefit. If we may raise one quibble it would be upon the question of the increase in capacity of the human stomach during the early weeks and months of life. A child of four weeks may take four ounces if opportunity serves, but that does not prove its capacity to deal with that amount. Nearly all young animals will eat more than they should, and dining "not wisely but too well" is a habit that may easily be contracted in very early life. However, the book as a whole is beyond praise, and the price brings it within the reach of all. We hope it may realise a very large sale.

### War Adventures and Experiences.

***My War Diary.*** By MARY KING WADDINGTON, author of "A Diplomatist's Wife," etc. (London: John Murray. Price 6s. net.)

THE story of how war broke over a world wholly unprepared can never stale, often as it has been told and re-told. Madame Waddington (widow of the late French Ambassador to England) has published letters and extracts from diaries written in Paris and other parts of France during these feverish months, and her narrative carries with it that air of actuality and continual shock of the unexpected which contemporary records alone have the secret of imparting. The narrative starts on August 1, 1914, and ends on a high note after the taking of Messines by the English. The earlier parts of the book are by far the most vivid. No pages are more thrilling than those which tell of the German occupation of the writer's home at Mareuil, the merciless devastation of the place, the cruel plunder, the brutal destruction of the peasants' property, clothing, and cherished little possessions. Madame Waddington took an active part in helping to restore something like order and comfort among the village population after the retirement of the enemy, and at all times was foremost in good work for the wounded. There are many interesting glimpses into hospital activities. The Japanese hospital at the Hôtel Astoria pleased the writer, particularly the nurses, "a funny collection of little yellow women, very polite and smiling and curtseying." "They say their touch is very light and they work more quickly than our nurses." She gives us an insight, too, into a side of hospital nursing not altogether unknown in this country, in the person of the "belle dame de la Croix Rouge," who "looked very nice in her *infirmière* dress and gave a great many orders, but wouldn't touch a wounded soldier." Finally, when things were being made hot for the "nurse," arrived the lady's husband, remonstrating, "Ma femme n'est pas habituée à retirer les chaussettes des pieds sales d'un soldat." And thereupon the authorities promptly closed her hospital and had the wounded carried off to the nuns.

The book is indispensable to those who would gain a view of the varied phases through which Paris has passed in these dreadful years.

**With the Scottish Nurses in Roumania.** By IVONNE FITZROY. With Illustrations and Map. (London: John Murray. Price 5s. net.)

DEDICATED to the memory of Elsie Maud Inglis, M.B., C.M., founder of the Scottish Women's Hospitals and C.M.O. of the London Units, this book appeals with peculiar force to all who have followed with admiration the too brief labours of this remarkable woman. The writer observes in her preface how "month after month her courage and endurance never flagged. Daily and hourly, in the very agony of suffering and death, she gave her life by inches." We find, however, too faint a record of Dr. Inglis's amazing struggle with circumstances in this vivid little volume. It is built up of extracts from a diary kept throughout the expedition, beginning August 1916 and ending June 1917, and has both the charm and the defects of this form of composition. Fresh and concise, the narrative carries with it the ineffaceable impression of the moment, but it is disjointed to a fault, and plunges on to new fields whenever the interest begins to gather. The Unit began work at Medjidia and we get fearful glimpses of their surgical labours under war conditions. Very soon they began to be exposed to the horror of constant bombardment from aeroplanes. Then a little later came the order to evacuate, and the tale of how this was done is given with splendid spirit. At Braila they toiled in a Roumanian hospital. Here the writer had "a ward of sixty patients, and all the windows are tight shut." At Reni in the heart of the winter the Scottish women went through great privations and terrible shortage of provisions—all bravely, even gaily, borne. "On one occasion during the worst of this terrible weather . . . when we went to carry the wounded in we found only two bodies frozen stiff under coverings that were just a sheet of ice. They had only about a quarter of a mile to travel to reach us." Through all the discouragement and depression of retreat Miss Fitzroy and her companions found time to laugh a little, chiefly at themselves and their discomforts. "One of the patients was heard to remark the other day, 'The Russian sisters are pretty, but they are not good; the English sisters are good, but they are not pretty.' We are very cast down." And so to the end, which lands the party at Aberdeen. We found it very difficult to lay down the book.

